# Obesity modulates the association between sleep apnea treatment and CHI3L1 levels but not CHIT1 activity in moderate to severe OSA: an observational study

**DOI:** 10.1007/s11325-018-1731-6

**Published:** 2018-10-11

**Authors:** Unnur Dilja Teitsdottir, Erna Sif Arnardottir, Erla Bjornsdottir, Thorarinn Gislason, Petur Henry Petersen

**Affiliations:** 1 Faculty of Medicine, Department of Biochemistry and Molecular Biology, Biomedical Center, University of Iceland, Reykjavik, Iceland; 2 Faculty of Medicine, University of Iceland, Reykjavik, Iceland; 3 Sleep Department, Landspitali - The National University Hospital of Iceland, Reykjavik, Iceland; 4 Reykjavik University, Reykjavik, Iceland

**Keywords:** Obstructive sleep apnea, Positive airway pressure, Obesity, Chitinase-3-like protein 1, Chitotriosidase

## Abstract

**Purpose:**

The inflammatory markers chitinase-3-like protein 1 (CHI3L1) and chitotriosidase (CHIT1) have both been associated with cardiovascular complications. The aim of this preliminary observational study was to assess the roles and interaction of obstructive sleep apnea (OSA) severity and body mass index (BMI) with plasma CHI3L1 levels and CHIT1 activity in patients with moderate to severe OSA. The second aim was to assess the roles and interaction of positive airway pressure (PAP) treatment and BMI on the expression of the same proteins.

**Methods:**

The study included 97 OSA patients with an apnea–hypopnea index (AHI) ≥ 15 and full usage of PAP treatment after 4 months. Plasma CHI3L1 levels and CHIT1 activity were measured before and after treatment.

**Results:**

Multiple linear regression analysis demonstrated an independent association of BMI on CHI3L1 levels (*p* < 0.05) but not on CHIT1 activity. The OSA severity markers (AHI and oxygen desaturation index) did not independently or in interaction with BMI levels associate with CHI3L1 levels or with CHIT1 activity (*p* > 0.05). A two-way repeated measures ANOVA revealed a significant interaction between PAP treatment effect (before vs. after) and BMI groups (< 35 kg/m^2^ vs. ≥ 35 kg/m^2^) on CHI3L1 levels (*p* = 0.03) but not on CHIT1 activity (*p* = 0.98).

**Conclusions:**

Obesity independently associated with CHI3L1 levels. Association between OSA severity and CHI3L1 levels or CHIT1 activity (independent of or dependent on obesity level) could not be confirmed. However, decrease was observed in CHI3L1 levels after PAP treatment in severely obese OSA patients but not in those less obese.

**Electronic supplementary material:**

The online version of this article (10.1007/s11325-018-1731-6) contains supplementary material, which is available to authorized users.

## Introduction

Obstructive sleep apnea (OSA) is a common disorder characterized by periodic reduction (hypopnea) or cessation (apnea) of airflow due to narrowing of the upper airway during sleep. OSA can effectively be treated with positive airway pressure (PAP) treatment [1]. Untreated OSA patients are at increased risk of developing cardiovascular complications such as hypertension, stroke, and cardiovascular disease [2].

Obesity is an important risk factor for both OSA and cardiovascular events. Obesity and OSA also activate similiar biological pathways of sympathetic activity, oxidative stress, and low-grade systemic inflammation [3]. It has therefore been a challenge separating the independent roles of OSA on cardiovascular disorders from those of obesity. It is likely that the role of OSA in cardiovascular disease is to some degree dependent on obesity. In our previous publications [4–6], we have found a moderating effect of obesity on how OSA affects both inflammatory-related protein levels, with the largest effect of OSA in severely obese patients with body mass index (BMI) ≥ 35 kg/m^2^. The concept that interaction between OSA and obesity increases inflammatory response could shape the design of future treatment clinical trials [3]. The discovery of biomarkers measuring this interaction effect is therefore of importance as it has the potential of providing important information about treatment response.

Chitinase-3-like protein 1 (CHI3L1, also known as YKL-40) and chitotriosidase (CHIT1) are proteins expressed in response to inflammation and have both been associated with cardiovascular disorders [7]. CHI3L1 is a 40-kD glycoprotein of the glycosyl hydrolase 18 family, secreted by activated macrophages, chondrocytes, neutrophils, and synovial cells [8]. The protein is one of four inactive chitinases that bind to chitin, but has no chitinase activity [9]. Elevated CHI3L1 levels have been associated with aging [10], degree of obesity [11], diabetes, and cardiovascular complications including atherosclerosis, acute myocardial infarction, and coronary artery disease [12]. A few studies [13–16] have also linked increased CHI3L1 levels to the presence and severity of OSA, although more research is still needed. CHI3L1 is secreted locally at the site of inflammation and not in response to systemic inflammation like some inflammatory markers (e.g., C-reactive protein [CRP]), indicating it could be of additional clinical use [17]. As an example, one study investigated the influence of statin treatment on CHI3L1 and high-sensitivity CRP levels in patients with stable ischemic heart disease (IHD) [18]. The statin-induced reduction of the two inflammatory markers was much more prominent for CHI3L1 than CRP, indicating difference between the two biomarkers in relation to their ability to monitor the inflammatory response in patients with IHD.

CHIT1 is a 50-kD mammalian chitinase of the same family. Unlike CHI3L1, CHIT1 is an active enzyme, mainly secreted by active macrophages and epithelial cells [19]. Previous studies have demonstrated that high CHIT1 activity is associated with Gaucher’s disease, atherosclerosis, and type 2 diabetes [20]. Very little is known about the relationship between CHIT1 activity and sleep. One previous study [21] linked CHIT1 activity to insufficient sleep in mice and rats. To our best knowledge, only one study has investigated the relationship between CHIT1 and OSA. Tamanaha et al. [22] tested whether plasma CHIT1 activity was related to the presence and severity of OSA, with results not indicating a significant association. Similarly, little is known about the relationship between CHIT1 and obesity. Alanbay et al. [23] found no significant correlation between CHIT1 activity and BMI.

No studies are available assessing the effect of PAP treatment on change in CHI3L1 levels or CHIT1 activity. The aim of this preliminary study was to evaluate the roles and interaction of OSA severity (AHI, oxygen desaturation index [ODI]), and obesity (BMI) on CHI3L1 levels and CHIT1 activity in a clinical cohort of patients with moderate to severe OSA (AHI ≥ 15). The second aim was to assess the roles and interaction of positive airway pressure (PAP) treatment and BMI on the expression of the same proteins.

## Methods

### Participants

A subset of 97 participants were selected from a larger 284 patients study [24]. All the participants had been diagnosed with OSA in Iceland and referred for PAP treatment to the Landspitali—The National University Hospital in Iceland from February 2010 to December 2013. The initial diagnosis of OSA was defined by AHI ≥ 15 events/h and oxygen desaturation index (ODI) ≥ 10 events/h. When sleep studies were rescored, there were however some subjects (*n* = 9) who had AHI between 10 and 15 events/h but they were not excluded from the study. More than 90% of approached subjects agreed to participate. Selection criteria for the subset used in this study were as follows: (1) participation in a sleep study before starting PAP treatment with a recorded apnea–hypopnea index (AHI) and oxygen desaturation index (ODI). All participants had moderate to severe OSA (defined as AHI ≥ 15) as that was a requirement for PAP treatment; (2) having blood samples taken while untreated and at follow-up; (3) used PAP treatment for ≥ 20 days and ≥ 4 h per day on average for the previous 4 weeks; and (5) completion of a 4-month follow-up as of May 13, 2014. This study was approved by the National Bioethics Committee and the Data Protection Authority of Iceland (10-048). Written consent was obtained from all subjects.

### Questionnaires and measurements

A core questionnaire on sleep, health, and medication use was answered by subjects at baseline and at a 4-month follow-up (e.g., if they had been diagnosed with hypertension, diabetes or cardiovascular disease, or other diseases by a physician). Height and weight were measured for all participants with the same instruments (a ruler and a scale), after having removed shoes as well as objects from clothes. Blood was drawn in the morning from the antecubital vein after overnight fasting, both at baseline from untreated participants and at follow-up. Prior to referral for PAP treatment, all subjects had a type 3 sleep study with a T3 device (Nox Medical, Reykjavik, Iceland), an Embletta type 3 portable monitor, or an Embla 12-channel system (Natus Medical Inc., San Carlos, CA, USA). Type 3 portable monitors were used for home sleep apnea testing (HSAT) as this is the clinical practice in Iceland as it is in many other countries, e.g., in Europe [25], and accepted by the AASM in the USA [26]. The study therefore lacks electroencephalographic recording and assessment of arousals. However, the validity of the Nox T3 portable monitoring system for assessing sleep-disordered breathing has been shown when compared with polysomnography with similar AHI and ODI levels [27, 28]. Also, the respiratory scoring rules used (requiring 4% oxygen desaturation for hypopnea events) do not require arousals to assess hypopneas. The same signals were recorded on all studies. Nasal airflow was recorded through a cannula. Chest and abdominal movements were measured using respiratory inductance plethysmography belts. Pulse and oxygen desaturation were measured by a finger probe oximeter based on a four-beat exponential average (Nonin Medical Inc., Plymouth, MN, USA). Body position and activity were measured using sensors situated on the chest. The sleep studies were scored by trained sleep technologists. Studies had to have ≥ 4 h of scorable oxygen saturation and more than two out of three respiratory traces: cannula flow, thorax and respiratory inductive plethysmography belts. Total analysis time was assessed based on questionnaires and the sleep technologist’s review of the study. Sleep studies were scored in accordance with the American Academy of Sleep Medicine (AASM) 2007 manual [29], using the recommended hypopnea classification requiring a ≥ 30% drop in respiratory flow for ≥ 10 s with ≥ 4% oxygen desaturation.

### PAP use

PAP adherence at follow-up was measured objectively by downloading the mask-on time stored by the PAP unit in the previous 4 weeks (S8 machines, ResMed, San Diego, CA, USA). Participants who used PAP for ≥ 20 days and ≥ 4 h per day on average for the previous 4 weeks were considered full users.

### Biomarker assessment

Blood was collected in BD vacutainers containing EDTA (BD, Franklin Lakes, NJ, USA), gently inverted 8–10 times, and placed on ice. Within 1 h of collection, the sample was spun for 15 min at 1790*g* in a refrigerated centrifuge. The samples were kept on ice during aliquoting. After separation, the plasma samples were stored at − 80 °C.

The CHI3L1 levels in human plasma samples were measured using a commercially available Sandwich Elisa Duoset from R&D systems (cat. DY2599, Minneapolis, MN). The assays were run in 96-well plates (Nunc 442404, MaxiSorp) using 100 μl of plasma (1:800 dilution) as per manufacturer’s instructions. For standards, human recombinant CHI3L1 protein was used at concentrations ranging from 31.25 to 2000 pg/ml. All samples were measured in duplicates.

Chitotriosidase enzyme assay was based on the method described by Hollak et al. [30] with minor modifications. Briefly, chitotriosidase activity was determined by incubating 5 μl of plasma with 100 μl of 22 mol/l fluorogenic substrate 4-methylumbelliferyl β-d-*N*,*N*′,*N*′′-triacetylchitotrioside (Sigma M5639) in 0.1 M/0.2 M citrate–phosphate buffer (pH 5.2) for 15 min at 37 °C. The reaction was stopped with 200 μl of 1 M glycine–NaOH buffer (pH 10.6) by mixing at room temperature. The substrate hydrolysis by chitotriosidase produces the fluorescent molecule 4-methylumbelliferone, which was quantified with a fluorometer (Spectramax M3 instrument), excitation at 360 nm and emission at 450 nm, and compared with a standard 4-methylumbelliferone (Sigma M1508) calibration curve. Plasma chitotriosidase activity was measured in triplicates. Inter-assay coefficient of variation (CV) for both CHI3L1 and CHIT1 was within 20% and intra-assay CV was within 10%.

### Statistical analysis

Descriptive group comparisons were performed using one-way analysis of variance and chi-square tests for continuous and categorical variables, respectively. CHI3L1 and CHIT1 were natural log-transformed for all analyses (based on assessment of residual error distribution, thereby permitting parametric analysis). The strengths of linear associations among biomarker values (CHI3L1 levels, CHIT1 activity), BMI, and OSA severity were assessed using Pearson’s correlation and multiple linear regression. Paired *t* test was used to compare the means of biomarker values before and after PAP treatment. Independent *t* test was used to compare the means of biomarker values between men and women. A two-way repeated measures ANOVA with interaction effects was performed to evaluate interaction effects between PAP treatment and BMI groups on biomarker values. All statistical analyses were performed using SPSS v.23.0 (IBM Corp., Armonk, NY, USA).

To avoid extrapolation beyond the scope of the data, sensitivity analyses were performed excluding those participants who had OSA severity not found in the other BMI groups [3]. All of the analyses led to the same conclusions as the original ones, and therefore all participants were used for the final analyses of the study.

## Results

### Baseline clinical and biochemical characteristics

Clinical and biochemical characteristics of OSA patients at baseline, both overall and within different BMI categories, are presented in Table 1. Severely obese subjects (BMI ≥ 35 kg/m^2^) had more severe OSA (higher AHI and ODI) as well as higher levels of CHI3L1, compared to patients with lower BMI (< 30 kg/m^2^ and 30–35 kg/m^2^). Furthermore, OSA patients within the highest BMI group also had a higher prevalence of type 2 diabetes compared to the other groups.

**Table 1:** Demographics and sleep-disordered breathing data at baseline in the whole cohort and by different BMI levels

	All *n* = 97	BMI < 30 *n* = 26	BMI 30–35 *n* = 37	BMI ≥ 35 *n* = 34	*p* ^a^
Age (years)	54.4 ± 10.5	56.4 ± 10.2	55.6 ± 10.0	51.6 ± 11.0	0.14
Males (%)	74.2	80.8	78.4	64.7	0.28
BMI (kg/m^2^)	33.9 ± 5.6	27.9 ± 1.5	32.4 ± 1.4	40.2 ± 4.1	N/A
Sleep-disordered breathing					
AHI (events/h)	39.0 ± 22.3	33.8 ± 16.2	34.7 ± 18.9	47.7 ± 27.2	0.02
ODI (events/h)	32.7 ± 21.3	26.8 ± 15.2	26.9 ± 18.2	43.7 ± 24.5	< 0.001
Medical history					
Hypertension (%)	66.7	54.2	79.4	62.5	0.11
Cardiovascular disease (%)	3.3	4.0	0.0	6.1	0.37
Stroke (%)	12.6	19.2	10.8	8.8	0.45
Type 2 diabetes (%)	14.6	12.0	5.4	26.5	0.04
Biomarker levels/activity					
CHI3L1 (ng/ml)^b^	3.9 ± 0.5	3.8 ± 0.5	3.8 ± 0.5	4.1 ± 0.5	0.03
CHIT1 (nmol/ml/h)^b^	3.2 ± 0.9	3.0 ± 1.1	3.3 ± 0.6	3.2 ± 1.1	0.49

Data are presented as mean ± standard deviation or % where indicated

^a^One-way analysis of variance for continuous variables and Pearson’s chi-square test for percentages for comparison between groups

^b^Exponentiated mean and 95% confidence interval determined from log-transformed values (i.e., geometric mean)

*BMI*, body mass index; *AHI*, apnea-hypopnea index; *ODI*, oxygen desaturation index; *CHI3L1*, Chitinase-3-like protein 1; *CHIT1*, Chitotriosidase

The range in values for AHI was between 15 and 116 events/h; for ODI, between 4 and 113 event/h; and for BMI, between 24.8 and 53.3 kg/m^2^.

### Association between OSA severity and obesity with chitinase levels and activity

**Fig. 1:**
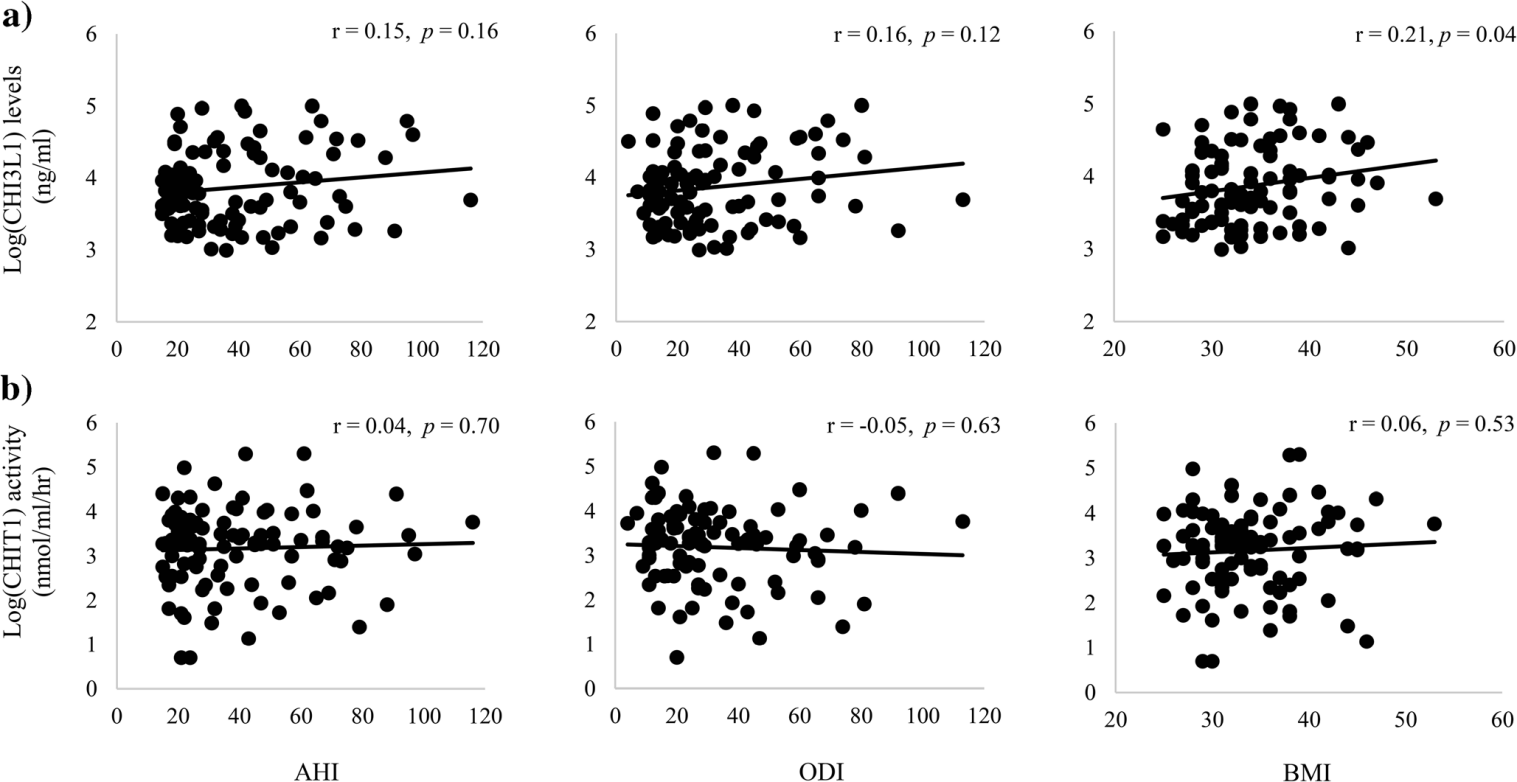
Pearson’s correlation between apnea–hypopnea index (AHI), oxygen desaturation index (ODI), BMI (body mass index) and CHI3L1 levels (**a**) and CHIT1 activity (**b**)

The correlation between chitinases and OSA severity markers (AHI, ODI) as well as obesity levels (BMI) was assessed. BMI was positively correlated with CHI3L1 levels (*r* = 0.21, *p* = 0.04) but not with CHIT1 activity (*r* = 0.06, *p* = 0.53). For OSA severity markers, a tendency for a weak positive correlation was observed with CHI3L1 levels, although not significant, and no relationship was found with CHIT1 activity (Fig. 1).

The relationships between the OSA severity markers and the chitinases were also assessed within the three different BMI categories. An indication of a specific pattern appeared for CHI3L1 levels. Although all insignificant (*p* > 0.05), both OSA severity markers correlated negatively with CHI3L1 levels within the lower BMI groups (< 30 kg/m^2^ and 30–35 kg/m^2^) but positively within the highest BMI group (BMI ≥ 35 kg/m^2^, Fig. S1a and Fig. S2a in supplement). No effect between OSA severity and CHIT1 activity was found when assessed within the three the same BMI categories (Fig. S1b and Fig. S2b in supplement).

To further evaluate how the level of BMI affects the relationship between OSA severity and chitinases, multiple linear regression analysis with interaction was performed (Table 2). The analysis demonstrated independent association of BMI and age with CHI3L1 levels. Significance (*p* < 0.05) was reached, independent of AHI (model 1) or ODI (model 2) being included in the analysis. Neither AHI nor ODI associated independently with CHI3L1 levels and the interaction between the markers and BMI was also non significant (*p* > 0.05). No independent association was found with CHIT1 activity.

**Table 2:** Multiple linear regression models, predicting plasma CHI3L1 levels and CHIT1 activity

		Log (CHI3L1) levels (ng/ml)			Log (CHIT1) activity (nmol/ml/h)		
		Standardized β (95% CI)		*p*	Standardized β (95% CI)		*p*
Model 1	AHI	0.12	(− 0.09–0.33)	0.27	0.01	(− 0.22–0.24)	0.94
	BMI	0.24	(0.03–0.45)	0.026	0.06	(− 0.17–0.28)	0.63
	BMI× AHI	0.01	(− 0.21–0.22)	0.94	0.06	(− 0.17–0.29)	0.60
	Age (years)	0.32	(0.13–0.52)	0.002	0.08	(− 0.13–0.28)	0.48
Model 2	ODI	0.14	(− 0.08–0.36)	0.21	− 0.10	(− 0.34–0.13)	0.40
	BMI	0.23	(0.01–0.44)	0.047	0.12	(− 0.12–0.35)	0.34
	BMI× ODI	0.01	(− 0.21–0.23)	0.899	0.06	(− 0.18–0.29)	0.65
	Age (years)	0.34	(0.14–0.54)	0.001	0.08	(− 0.13–0.29)	0.46

### The effect of PAP treatment on chitinase levels and activity

**Fig. 2:**
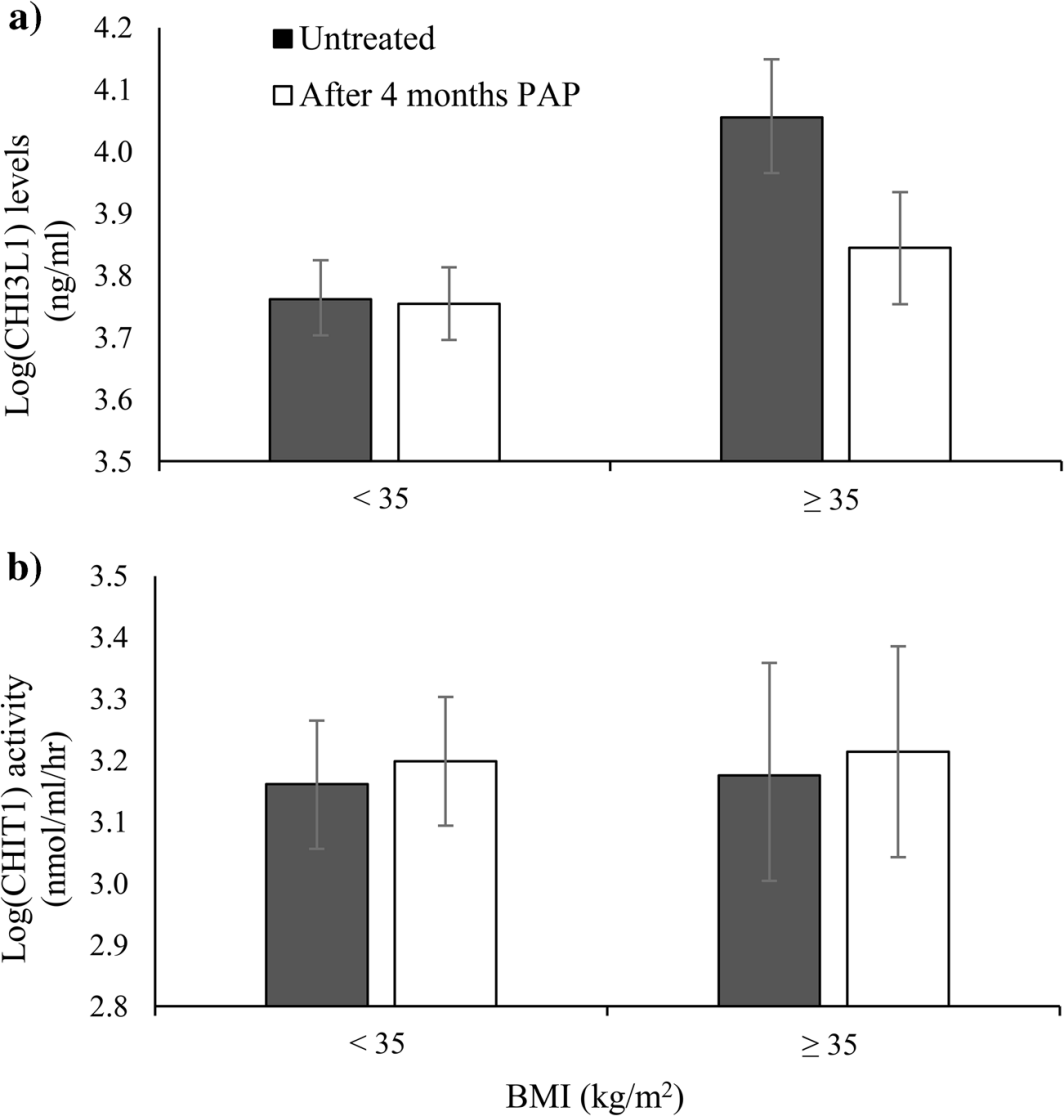
Mean ± SEM CHI3L1 levels (**a**) and CHIT1 activity (**b**) before and after PAP treatment by two BMI groups

Overall, no significant differences in CHI3L1 levels (3.87 ± 0.05 vs. 3.79 ± 0.05, *p* = 0.08) and CHIT1 activity (3.17 ± 0.09 vs. 3.20 ± 0.09, *p* = 0.08) were found among OSA patients before and after PAP treatment. There was a trend towards an interaction between treatment and BMI, although the interaction effect did not reach significance (*p* = 0.08, Fig. S3 in supplement). For an increased power, we also ran the analysis by combining the two lower BMI groups. Figure 2 presents differences in CHI3L1 levels and CHIT1 activity before and after PAP treatment when stratified by two BMI groups (< 35 kg/m^2^ and ≥ 35 kg/m^2^). A two-way repeated measures ANOVA revealed a significant interaction (*p* = 0.028) between the two factors for CHI3L1 levels showing a significant decrease in CHI3L1 levels for OSA patients with PAP treatment with BMI ≥ 35 only, not those with BMI < 35 (Fig. 2a). No significant interaction between PAP treatment and BMI groups (*p* = 0.976) was found for CHIT1 activity (Fig. 2b).

Four additional repeated measures ANOVA analyses were also performed, with each analysis excluding a different group of patients from the study, to assess the potential confounding effects of other diseases. Each analysis excluded patients with hypertension, cardiovascular disease, stroke, or type 2 diabetes. Interaction effects between PAP treatment and the two BMI groups reached significance for CHI3L1 levels (*p* < 0.05) but not for CHIT1 activity, independent of which patient group was excluded. This is in accordance with the primary analysis. No significant differences were found between men and women in CHI3L1 levels at baseline (3.86 ± 0.06 vs. 3.87 ± 0.10, *p* = 0.99) or after treatment (3.78 ± 0.06 vs. 3.82 ± 0.10, *p* = 0.72). Despite this lack of difference, sensitivity analysis was performed including men only (*n* = 72). The interaction effect for CHI3L1 levels showed a similar trend as for the main analysis but did not reach significance (*p* = 0.20, Fig. S4 in supplement).

## Discussion

The results of this study indicate that effects of PAP treatment on CHI3L1 levels depend on the level of obesity (measured by BMI) in a population of patients with moderate to severe OSA. Obesity did also positively associate with the concentration of the same protein. The effect of OSA severity on CHI3L1 levels, with and without the degree of obesity, was not significant indicating that the AHI and ODI as markers of OSA severity may not be appropriate to assess the impact of OSA on CHI3L1 levels in the same patient populations. Furthermore, the effect of PAP treatment, degree of obesity, and OSA severity did not associate with CHIT1 activity.

Few recent studies have demonstrated an independent association of both the presence and severity of OSA on serum CHI3L1 levels [13–16]. Our results are not in accordance with these previous studies as an independent relationship between OSA severity and CHI3L1 levels could not be confirmed. A possible explanation is that our sample did only include patients with moderate to severe OSA, not those with mild OSA and healthy controls. Our results, on the other hand, confirmed an independent, positive association of obesity and age with CHI3L1 levels, which has also been reported in earlier studies [10, 11, 31].

Obesity is an important risk factor in the pathogenesis of OSA [32–34]. As obesity and OSA tend to coexist, they can also independently affect similar biological pathways such as oxidative stress and inflammation [35–38]. Previous studies have indicated an accumulation of activated macrophages and other immune active cells in visceral adipose tissue from obese subjects as possible sources of inflammatory cytokines, determining a link between obesity and low-grade inflammation [39, 40]. It could therefore be hypothesized that the effect of OSA is amplified by the increased number of inflammatory cells in fat in the most obese patients [41]. Arnardottir et al. [4] demonstrated, in a large study based on the Icelandic Sleep Apnea Cohort, that the independent association of OSA with levels of pro-inflammatory cytokines interleukin-6 (IL-6), and CRP depended on the degree of obesity. These results were furthermore confirmed in a long-term follow-up [5] where the increase of interleukin-6 was prevented by PAP treatment, but only among severely obese patients (BMI ≥ 35 kg/m^2^). CHI3L1 has been identified as a major protein expressed in macrophages among stromal vascular fraction cells in human visceral adipose tissue [42]. When assesing the role of PAP treatment and obesity on CHI3L1 levels, our results came to similar conclusions with these studies. Change in levels of the protein after PAP treatment did depend on degree of obesity. Reduction in CHI3L1 levels was found among severely obese patients (BMI ≥ 35 kg/m^2^) after treatment but not in those less obese (BMI < 35 kg/m^2^).

An interaction effect did not appear between PAP treatment and obesity regarding CHIT1, as there were no significant changes in activity after treatment when analyzed by different BMI categories. Very few studies have examined the relationship between CHIT1 enzyme activity and sleep. A previous study by Lungato et al. [21] showed association between CHIT1 plasma levels and paradoxical sleep deprivation in adult male mice and rats. They found a significant increase in CHIT1 plasma levels when the animals were subjected to paradoxical sleep deprivation for 72 h compared to controls. Because sleep has an important role for the maintenance and efficiency of the immune system [43, 44], their data suggested a relationship between sleep and macrophage response, where CHIT1 activity could possibly serve as a marker for insufficient sleep. To our best knowledge, only one study has investigated the relationship between OSA and CHIT1. Tamanaha et al. [22] tested whether plasma CHIT1 activity was related to the presence and severity of OSA in a Brazilian cohort, where the results did not indicate a significant association. Our results are in accordance with those results: no association was measured between OSA severity and CHIT1 activity. The relationship did not appear to depend on obesity either, with no specific interaction effect appearing. To our best knowledge, it has not been tested if the enzyme is expressed by macrophages and endothelial cells in the visceral adipose tissue.

There are a few major limitations of the present study. First, this is an observational study, not a randomized controlled trial, with all participants receiving full PAP treatment. Therefore, our findings are preliminary and have to be interpreted with caution. Second, this was a study with a relatively small number of OSA patients and present findings need to be validated in a larger study. The sample did not include non-OSA controls or mild OSA patients and the limited variability in the lower end of OSA severity and BMI could underestimate associations between the studied variables. The sample was also relatively homogeneous with Caucasian and mostly male participants. The results might therefore not generalize to other more ethnically diversed populations or across gender. No difference in serum CHI3L1 levels between males and females have though been found in previous studies [45]. Third, we did not have other measurements of obesity like visceral fat and waist circumference, change in BMI during the 4-month treatment period, or concentrations of other pro-inflammatory mediators, which may provide valuable information on a possible role of CHI3L1 and CHIT1 in the progression of OSA. However, our previous paper assessing the role of different fat measures in inflammatory biomarkers showed that total fat measures such as BMI were more highly associated with inflammatory levels than visceral fat levels per se [4]. Fourth, with multiple sensitivity analyses (excluding each disease), there is an increased risk of false positive results (type 1 error). By replicating the study in a larger sample, more information could be fitted into the statistical model, decreasing the risk of this type of error. Also, with a larger sample, estimation of coefficients will be more precise. Finally, residual confounding cannot be fully excluded as confounding factors were assessed with crude precision (e.g., having a condition vs. not having a condition).

In conclusion, this is the first paper to assess the effect of PAP treatment on CHI3L1 levels and CHIT1 activity in OSA patients. The results show a decrease in CHI3L1 levels after 4 months of full PAP treatment in severely obese OSA patients (BMI ≥ 35 kg/m^2^), but not in those less obese (BMI < 35 kg/m^2^). The same effect was not observed with CHIT1 activity. Obesity and age were independently associated with CHI3L1 levels but not with CHIT1 acitvity. Association between OSA severity and CHI3L1 levels/CHIT1 activity (independent of or dependent on obesity) could not be confirmed.

## Electronic supplementary material

ESM 1 (PDF 423 kb)
