# Supplementary material for: Obesity modulates the association between sleep apnea treatment and CHI3L1 levels but not CHIT1 activity in moderate to severe OSA: an observational study
Source: Sleep Breath. 2018 Oct 11;22(4):1101–9. doi: 10.1007/s11325-018-1731-6 (PMC6244533; doi:10.1007/s11325-018-1731-6)
Supplement: Supplementary file 1 — (PDF 423 kb) [file 11325_2018_1731_MOESM1_ESM.pdf]

Supplemental material

Obesity modulates the association between sleep apnea treatment and CHI3L1 levels but not CHIT1 activity.

Authors: Unnur Dilja Teitsdottir[1], Erna Sif Arnardottir[2,3], Erla Bjornsdottir[3,4], Thorarinn Gislason[2,3], Petur Henry Petersen[1]

Affiliations:

[1]Faculty of Medicine, Department of Biochemistry and Molecular Biology, Biomedical Center, University of Iceland, Reykjavik, Iceland.

[2]Faculty of Medicine, University of Iceland, Reykjavik, Iceland.

[3]Sleep Department, Landspitali - The National University Hospital of Iceland, Reykjavik, Iceland.

[4]Reykjavik University, Reykjavik, Iceland.

Corresponding author is Unnur Diljá Teitsdóttir, email: udt1@hi.is

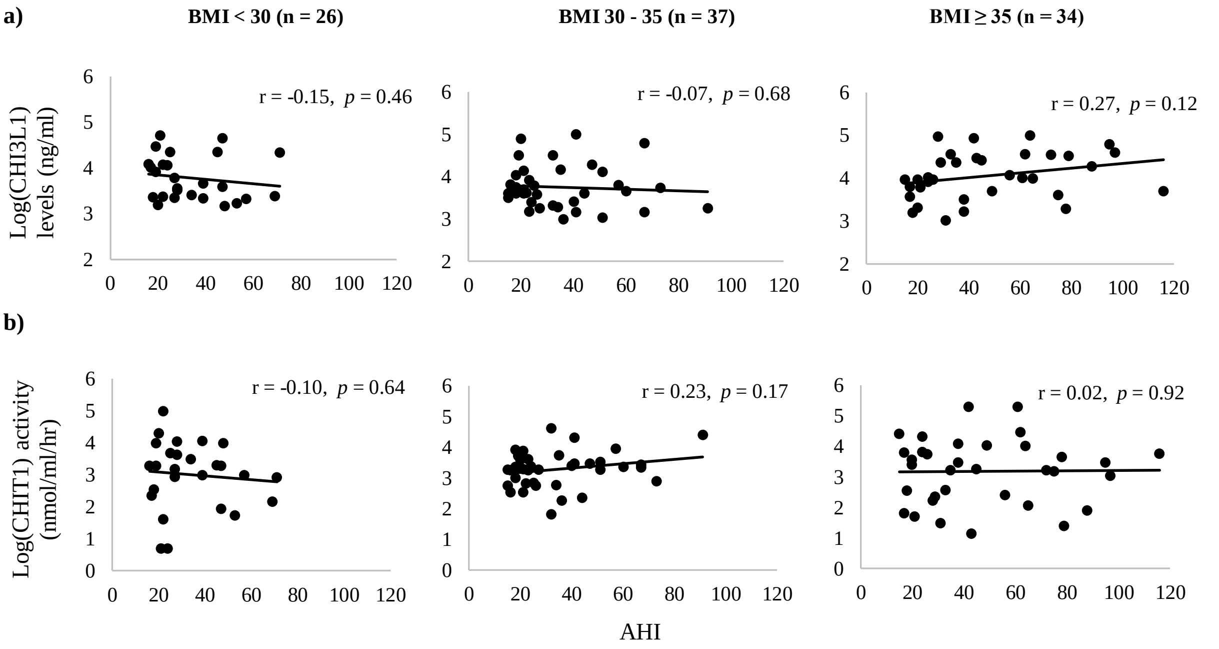

Fig. S1. Correlation between the apnea-hypopnea index (AHI) and a) CHI3L1 levels and b) CHIT1 activity within the 3 BMI categories.

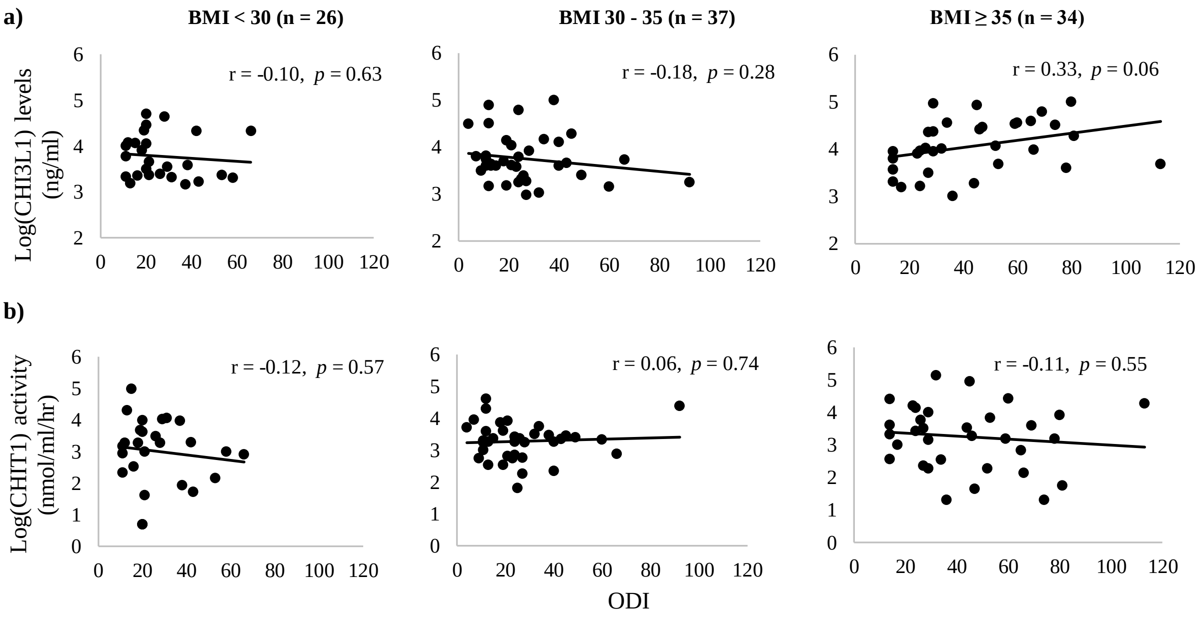

Fig. S2. Correlation between the oxygen-desaturation index (ODI) and a) CHI3L1 levels and b) CHIT1 activity within the 3 BMI categories.

a)

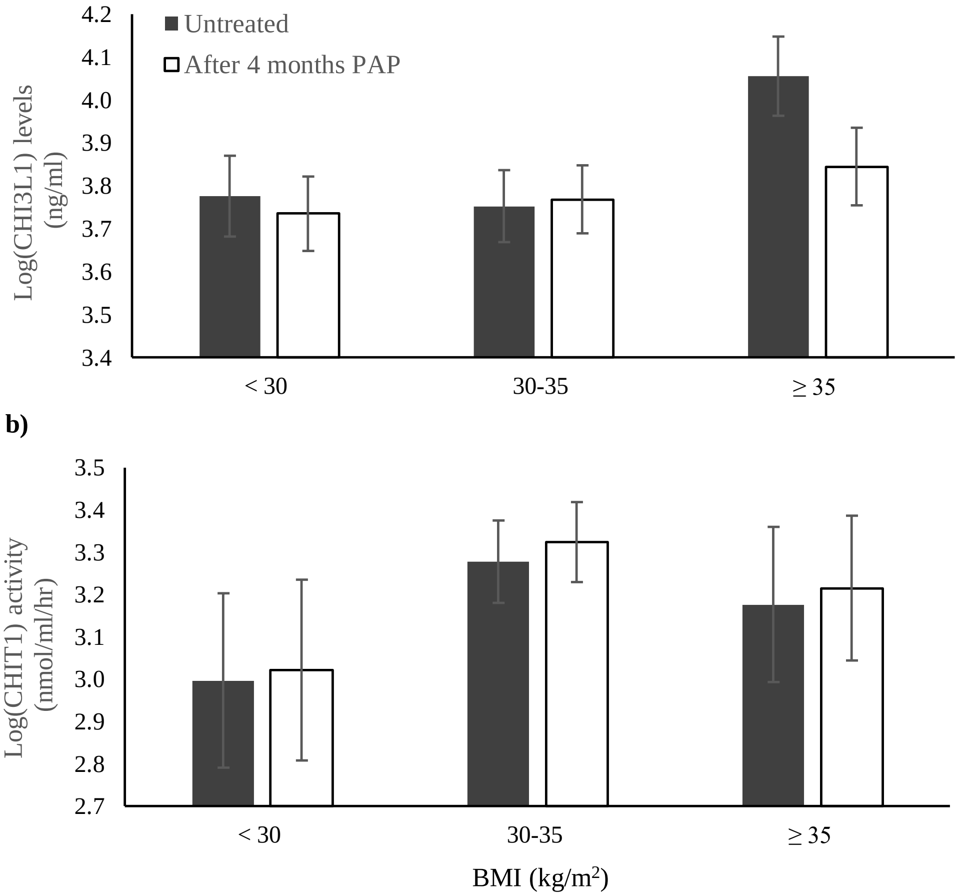

Fig. S3. Mean ± SEM a) CHI3L1 levels and b) CHIT1 activity before and after PAP treatment by three BMI groups.

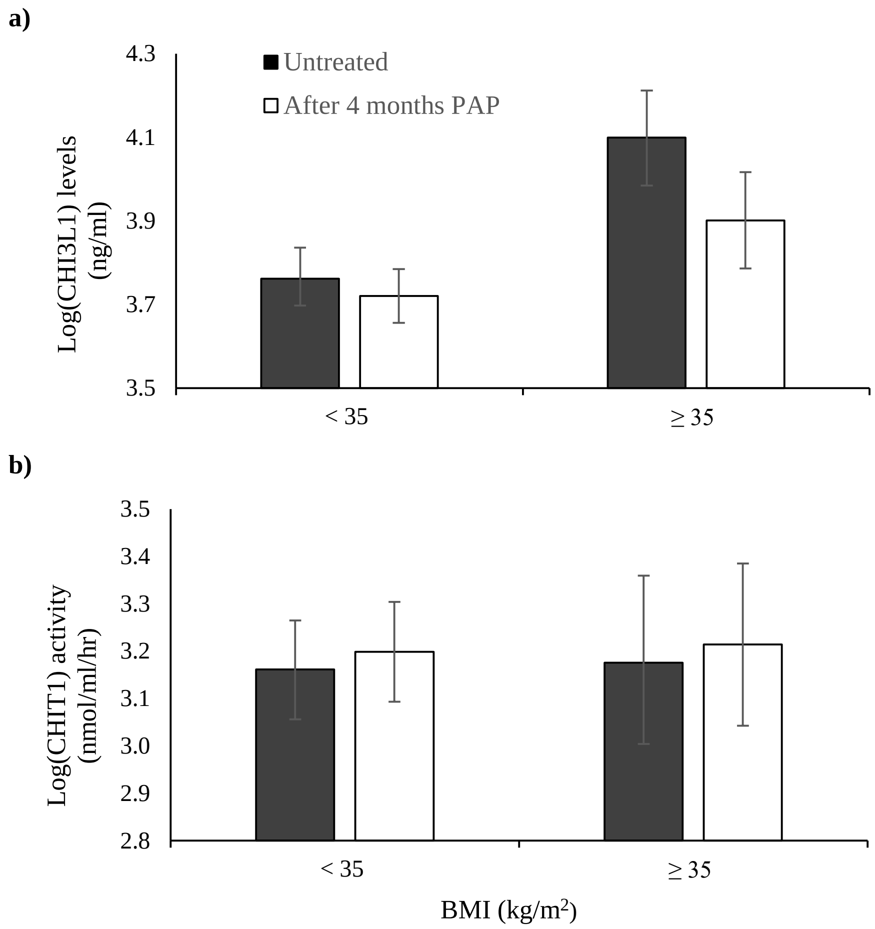

Fig. S4. Mean ± SEM a) CHI3L1 levels and b) CHIT1 activity before and after PAP treatment by two BMI groups, only including men (n=72) in the analysis.
